# Optical rectification and thermal currents in optical tunneling gap antennas

**DOI:** 10.1515/nanoph-2022-0278

**Published:** 2022-08-10

**Authors:** Marie Maxime Mennemanteuil, Mickaël Buret, Gérard Colas-des-Francs, Alexandre Bouhelier

**Affiliations:** Laboratoire Interdisciplinaire Carnot de Bourgogne CNRS UMR 6303, Université de Bourgogne Franche-Comté, 21000 Dijon, France

**Keywords:** optical gap antennas, optical rectification, photo-assisted transport, rectennas, thermo-current, thermo-voltage

## Abstract

Electrically-contacted optical gap antennas are nanoscale interface devices enabling the transduction between photons and electrons. This new generation of device, usually constituted of metal elements (e.g. gold), captures visible to near infrared electromagnetic radiation and rectifies the incident energy in a direct-current (DC) electrical signal. However, light absorption by the metal may lead to additional thermal effects which need to be taken into account to understand the complete photo-response of the devices. The purpose of this communication is to discriminate the contribution of laser-induced thermo-electric effects in the photo-assisted electronic transport. We show case our analysis with the help of electromigrated devices.

## 1 Introduction

Harvesting of electromagnetic energy has been fueling the quest for developing alternative technologies to compete with semiconductor-based photovoltaics. Amongst the different pretenders are metal-based rectifying antennas operating in the visible part of the spectrum [1, 2]. These nanodevices combine in a footprint commensurate with the incoming wavelength the receiving antenna and the rectifying element in the form of a tunnel gap. Several advantages speak for such monolithic integration of the antenna and the rectifier. The electromagnetic response of the gap provides an enhanced interaction with the incident light [3, 4]; the gap serves as a capture antenna. Then, the static electric polarity applied across the gap, geometrical and material asymmetries [5, 6], as well as the ultrafast transit time across the tunneling barrier [7] contribute to rectify high frequency radiation to an electrical DC signal. Hence, the gap acts as a rectifier for visible light [8–10]. This new family of devices found new application venues. Optical rectification occurring at the level of an individual nanoscale rectenna has been observed repeatedly by a direct illumination of the tunneling gap [11–14], enabling thereby on-chip detection of light signal [15–17].

However, rectification of the incident light may not be the only mechanism providing a photogenerated electrical signal. A portion of the light is inevitably absorbed by the metal parts, which lead to an increase of the system’s temperature. Expansion of the electrodes, thermo-induced voltages and currents are therefore likely to affect the conductance of the tunneling gap and contribute to a concurrent current flow summed with the rectified response. While combining different mechanisms for generating a current may be of advantage to increase the overall efficiency of the device, the temporal dynamics of thermal effects are many orders of magnitude slower than optical rectification and are thus limiting the attainable response bandwidth. These adverse thermal effects were preponderant in the context of photo-assisted scanning tunneling microscopy [18], but were generally discarded for planar devices on account of a better heat dissipation via the substrate [11, 12]. However, reports also demonstrated the occurrence of photothermal electrical responses in nanoscale junctions and constrictions [14, 19], [20], [21], [22], [23] as well as a sensitivity of thermo-electrical power to atomic gap configurations [24], and biasing polarity [23, 25]. There is therefore a need to complement and link this body of literature with experiments specifically addressing the contribution of thermal effects in the response of tunneling optical rectennas. To that aim, we analyse in this work the diverse parameters contributing to an electrical signals generated by a laser-illuminated optical rectenna fabricated by electromigration. We provide general guidelines to mitigate thermal contributions in this type of devices.

### 1.1 Fabrication procedure

**Figure 1:**
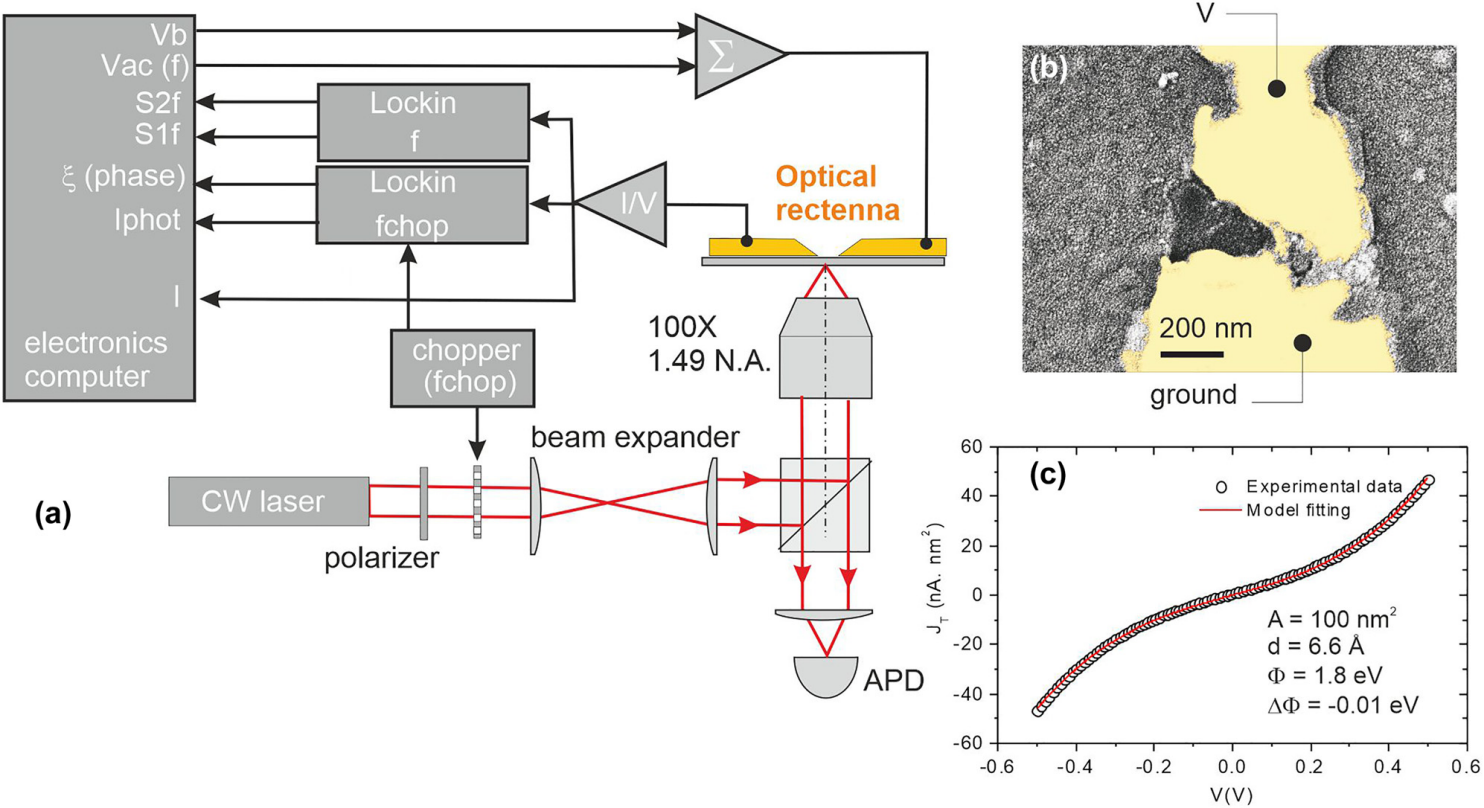
Electromigration and characterization. (a) Schematic of the experimental bench showing the optical excitation of the rectenna and the different electrical measurements used to analyse the current photogenerated by the device under test. (b) Scanning electron micrograph of a junction realized by electromigration. Au is represented by a yellow hue. (c) Typical output characteristic and a fit to the data. The extracted parameters are the gap distance *d*, the average barrier energy Φ, and its asymmetry ΔΦ.

At the heart of the optical rectennas discussed here is a sub-nanometer tunneling barrier separating two metal electrodes. For optical characterization purposes and ease of fabrication, we fabricate in-plane tunnel gaps by conducting a controlled electromigration [26] of two tapered Au electrodes bridged by a nano-constriction. These parts are realized by electron-beam lithography on a glass coverslip and subsequent thermal evaporation of a 3 nm thick Cr adhesion layer and a 60 nm thick Au layer. The typical dimensions of the constriction are 200 nm × 200 nm. Macroscopic contact electrodes used to electrically interrogate the device are then fabricated by standard optical lithography followed by the same metal evaporation processes. To electromigrate the constriction, we proceed with a methodology described elsewhere [27]. In brief, we apply a voltage *V* across the two contact electrodes and monitor the time evolution of the constriction’s dynamic conductance. The onset of electromigration is characterized by a rapid decay of the conductance *G*, and generally occurs for an applied voltage of about *V* = 2 V. To contain the catastrophic runaway of the process, *V* is immediately reduced. The amplitude of the voltage is then either slightly increased to trigger again the atomic rearrangement and thinning down of the constriction or left constant if Joule heating imparted by the current flowing in the device is sufficient to assist the electromigration. This process is cycled until the conductance reaches the tunneling regime characterized by *G* < *G*_0_, where *G*_0_ = 2e^2^/*h* = 77 μS is the quantum of conductance, e is the electron charge and *h* is Planck’s constant. Figure 1(a) sketches the experimental bench used to prepare and characterize the optical rectenna. The details of the experiment will be discussed later in the text.

A typical scanning electron micrograph of a junction is displayed in the false-color image of Figure 1(b). The contours of the broken section are ill-defined because of the stochastic nature of the electromigration. The rupture of the gold electrode does not necessarily occur at the constriction, but is slightly displaced toward the source electrode [27]. Note that for the purpose of electron imaging, a thin Au conductive layer has been deposited on the entire sample after being fully characterized. From a general point of view, we typically faced device-to-device variability, especially with regard to the gap morphology, the efficiency of the response and the sensitivity to thermal effects. Many devices were fabricated and tested and the general behavior discussed here was routinely observed in one form or another. We experienced difficulties at maintaining devices stable enough to withstand the whole range of electro-optical characterization discussed below. Nonetheless, and for the sake of consistency the results presented in the following sections were acquired from a single device, otherwise mentioned.

### 1.2 Electrical characterization

To have a complete picture of the rectenna’s operation, we start by electrically characterizing the output characteristic of the tunneling barrier. We treat the barrier in the framework of Simmons’ model of transport [28] and apply the methodology described by Dasgupta et al. [27]. In the limit of a small electron kinetic energy eV compared to the average barrier height Φ, the conductance dependence with the voltage bias *V* applied across the gap takes an analytical form [29]:

(1) $$G\left(V\right)=\frac{dJ}{dV}=G\left(0\right)\left(1-\frac{\Gamma \Delta \Phi }{16\Phi^{\frac{3}{2}}}V+\frac{9\Gamma^{2}}{128}V^{2}\right),$$

where ΔΦ = Φ_1_ − Φ_2_ represents the difference of the barrier height at the two sides of the gap, $\Gamma =4\sqrt{2m}d/3\hbar$ with *m* the electron mass and *d* the gap distance. *G*(0) writes [29]:

(2) $$G\left(0\right)=A\times 3.16\cdot 10^{-4}\frac{\sqrt{\Phi }}{d}\exp\left[-1.025d\sqrt{\Phi }\right].$$

*A* is the effective area of the junction in nm^2^, *d* is in Å, *V* is in V, and Φ is in eV.

The current density flowing through the barrier *J*(*V*) = *I*(*V*)/*A* is thus

(3) $$\begin{array}{cc} J\left(V\right) & =\overset{V}{\underset{0}{\int }}G\left(V\right)dV^{\prime }\\ & =\left(\frac{3.16\cdot 10^{-4}\sqrt{\Phi }}{d}V-\frac{2.7\cdot 10^{-5}\Delta \Phi }{\Phi }V^{2}\right.\\ & \;\left.+\frac{5.5\cdot 10^{-5}d\sqrt{\Phi }}{\Phi }V^{3}\right)\exp\left[-1.025d\sqrt{\Phi }\right]\; \end{array}$$

The set of parameters [*d*, Φ, ΔΦ] can be extracted by fitting the experimental output characteristic *I*(*V*) with Eq. (3) and fixing the junction’s area *A*. Figure 1(c) shows a typical output characteristic of an electromigrated junction together with a fit of the experimental data points using Eq. (3). *A* is not easily accessible experimentally considering the complex geometry of the gap (see Figure 1(b)). Here, we tentatively set *A* = 10 × 10 nm^2^. In an earlier report, we showed that *A* has a marginal influence on the value of the gap size [27]. In this example, the estimated gap is 6.6 Å and the barrier is weakly asymmetric with respect to the applied polarity (negligible ΔΦ). The extracted barrier height (Φ = 1.8 eV) is much smaller than the work function of Au, but is consistent with previous reports of reduced barrier height in similar systems [14, 27, 30], [31], [32]. From the electrical point of the view, there is no evidence that the Cr adhesion layer plays a role in the conduction properties.

### 1.3 Laser-induced signals

To interrogate the response of the device upon laser illumination, the electromigrated junction is placed on an inverted microscope equipped with a high numerical aperture (NA) oil immersion objective lens (NA = 1.49, 100×). A 785 nm constant-wave (CW) laser is focused to a diffraction-limited area by the objective. The focal spot is estimated at 260 nm (full-width-at-half-maximum). The sample is raster scanned through the focal region to reconstruct maps of the different optical and electrical responses of the rectenna. We use a transimpedance amplifier to measure the current at the output of the device. The total current *I* is the sum of the bias-induced tunnel current *I*_b_ and the photogenerated current *I*_phot_:

(4) $$I=I_{\text{b}}+I_{\text{phot}}$$

At *V* = 0, *I*_b_ is null and only *I*_phot_ exists. However, if the junction is externally biased, the contributors of the right hand side of the above expression are added. To extract *I*_phot_ from the total current regardless of the electrical biasing condition, we introduce a lock-in detection by chopping the laser beam at frequency of *f*_chop_ = 831 Hz. The chopper provides a reference signal to sync the lock-in amplifier.

**Figure 2:**
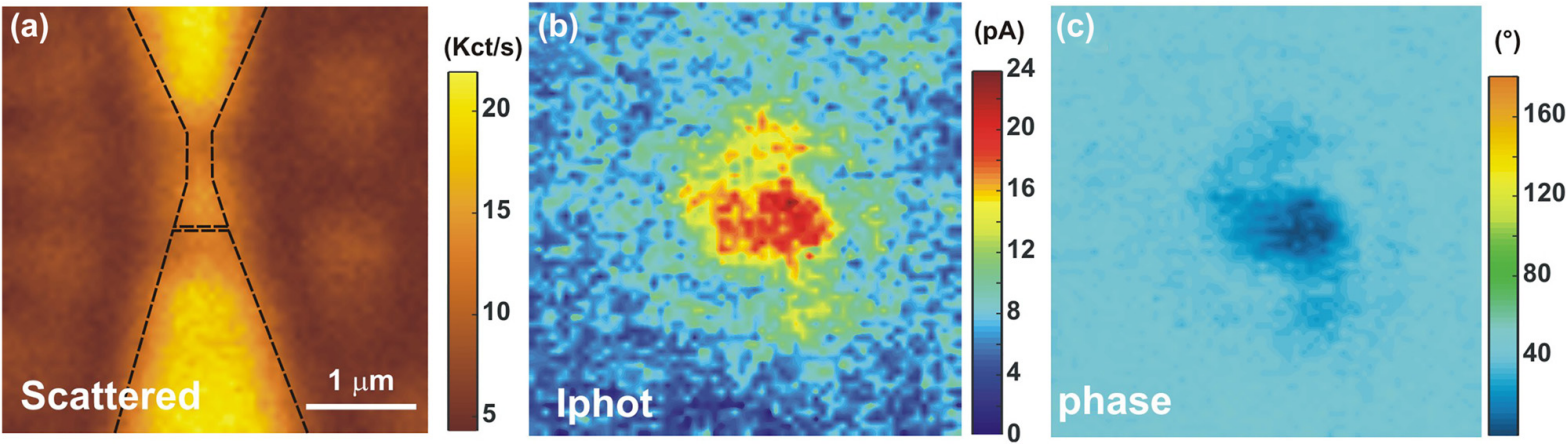
Spatially-resolved confocal maps. (a) Map of the back-scattered laser intensity. The outline of the tapered electrode, the constriction and the electromigrated region are marked by the dotted lines. (b) and (c) Maps showing simultaneously measured modulus |*I*_phot_| and phase *ζ* of the laser-induced current. No electrical bias is applied here: *V* = 0 V.

An example of the junction’s responses is illustrated in the confocal maps of Figure 2. Figure 2(a) is a map of the laser intensity partially back-reflected from the sample as it is scanned through the focus. The reflected laser intensity is recorded by an avalanche photodiode (APD) placed in a conjugate object plane of the microscope (see Figure 1(a)). This reflection map helps us to identify the device geometry because Au surfaces give a higher reflected laser signal. The incident polarization is orthogonal to the main axis of the system to maximize the image contrast of the back-scattered intensity. We fixed this polarization throughout the rest of the manuscript because it is difficult to predict an optimum polarization orientation considering the complex geometry of electromigrated gaps. In Figure 2(a), the electrodes and the position of the tunnel junction are approximately outlined by the dotted lines. The laser intensity at the focal spot is estimated at 486 kW cm^2^ and a density filter attenuates the reflected beam detected by the APD. We simultaneously record the modulus and phase outputs of the lock-in amplifier, |*I*_phot_| and *ζ*. These two signals are displayed in Figure 2(b) and (c) for *V* = 0, respectively. When the electromigrated gap overlaps the laser focus, there is a photocurrent of approximately 20 pA generated. The lateral extension of the response results from a convolution between the diffraction-limited area of the excitation spot and the capture cross-section of the responsive region, and perhaps a residual over or under focused position of the objective lens. The phase signal displayed in Figure 2(c) stays approximately in-phase (*ζ* = 0) whenever a photo-signal is detected. This indicates that the direction of the current remains constant regardless of the laser position. The question at this stage of the discussion is to identify the possible processes contributing to |*I*_phot_| when the laser overlaps the feedgap region of the device.

## 2 Contributing laser-induced processes

In the following sections we review the different mechanisms that could potentially contribute for generating a laser-induced current in the tunnel junction.

### 2.1 Optical rectification from a classical perspective

**Figure 3:**
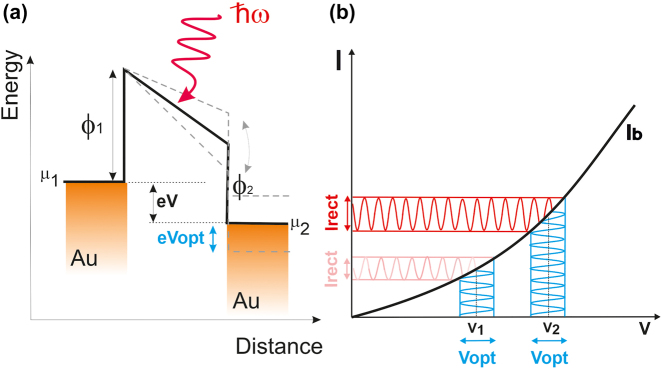
Interaction of the tunnel barrier with an electromagnetic wave. (a) Energy diagram picturing a tunnel barrier biased at illuminated by an electromagnetic field with a photon energy *ℏω*. The oscillation of the barrier at *ω* creates an optically-induced voltage *V*_opt_. *μ*_1_ and *μ*_2_ are the chemical potential of the two Au electrodes and *ϕ*_1_ and *ϕ*_2_ are the effective work functions. (b) Sketched output characteristics showing the amplitude of the rectified current *I*_rect_(*V*_opt_) for two biasing operating set-points *V*_1_ and *V*_2_.

Rectification of the electromagnetic field can be cast from classical concepts ruling the operating mode of microwave rectennas. An illustration of the energy landscape of a generic biased rectenna is depicted in Figure 3(a). A potential barrier between two metallic electrodes whose electrochemical potentials *μ*_1_ and *μ*_2_ are separated by an energy eV, undergoes additional oscillating potential *V*_opt_ in the presence of an incident radiation at an energy *ℏω*. Figure 3(b) illustrates the effect of the working point *V* of the device output’s characteristics on the amplitude of the rectified current. The nonlinear evolution of the *I*_b_(*V*) curve provides a handle to control the magnitude *I*_rect_ with the external static bias *V* for a given optical voltage *V*_opt_ produced at the gap.

Following a classical description of the rectification and neglecting for now any additional thermal contributions, the total bias at the terminal of the rectenna is

(5) $$V_{\text{rect}}\left(t\right)=V+V_{\text{opt}}\cos\left(\omega t\right)$$

The photo-assisted tunneling (PAT) current *I*_PAT_(*t*) induced by the optical potential *V*_opt_ can then be obtained by a Taylor expansion [1]:

(6) $$\begin{array}{cc} I_{\text{PAT}}\left(t\right) & =\overset{+\infty }{\underset{n=0}{\sum }}\frac{1}{n!}\frac{d^{n}I_{\text{b}}}{dV^{n}}{\left[V_{\text{rect}}\left(t\right)-V\right]}^{n}\\ & =\overset{+\infty }{\underset{n=0}{\sum }}\frac{1}{n!}\frac{d^{n}I_{\text{b}}}{dV^{n}}{\left[V_{\text{opt}}\cos\left(\omega t\right)\right]}^{n} \end{array}$$

Using the trigonometric relation $V_{\text{opt}}^{2}\cos^{2}\omega t=\frac{V_{\text{opt}}}{2}\left[1+\cos\left(2\omega t\right)\right]$ and similar relations developed at higher orders, the tunnel current *I*_PAT_(*t*) is described by the relation:

(7) $$I_{\text{PAT}}\left(t\right)=\overset{+\infty }{\underset{n=0}{\sum }}I_{n}\cos\;n\omega t$$

Considering only the term corresponding to the lowest order, the time averaged tunnel current through the nanojunction under light excitation and voltage *V* is written:

(8) $$I=I_{\text{b}}+\frac{1}{4}V_{\text{opt}}^{2}\frac{d^{2}I_{\text{b}}}{dV^{2}}=I_{\text{b}}+I_{\text{rect}}$$

The first term corresponds to the current produced by the voltage *V* applied to the rectenna while the second term corresponds to the additional current rectified by the device. This expression was used by Tu et al. [33] to analyse their experimental data on rectification at microwave frequencies. Equation (8) was also used by Bragas [34] and then Natelson [12] to identify and distinguish the contribution of the rectified current from the total current for rectennas illuminated in the visible spectral region.

Equation (6) assumes that the current flowing through the rectenna follows instantaneously the applied potential. However, this assumption is no longer valid when the period of the oscillations is comparable to the transit time of the electrons in the nanojunction. The classical description of the rectification process is therefore only applicable for devices operating at low frequencies or when the optical voltage *V*_opt_ created by the barrier oscillation is much lower than ℏ*ω*/*e*. Formally, the interaction of the rectenna with an optical field requires a quantum or semiclassical interpretation based on the photo-assisted tunneling [2, 9, 35]. We will see in the following of this study that electromigrated junctions have a sufficiently low nonlinearity with respect to ℏ*ω*/*e* to consider the classical model even in the presence of a high frequency excitation.

### 2.2 Thermal effects

When the laser illuminates the rectenna, and by extension its electrical feed-throughs, one should take into account the accompanying thermal effects promoted by the absorption of light. For subnanometer junctions, thermal expansion of the leads is likely to affect the magnitude of the tunneling current by closing the gap separating the two electrodes. This mechanism is particularly adverse in scanning tunneling microscopy [18] but was found to be negligible in device anchored to a substrate and illuminated with laser energy much lower than the material’s interband transitions [11]. Another contributor is induced by a temperature gradient that may exist between the two sides of the tunneling junction. A difference in temperature Δ*T* introduces an asymmetric electronic distribution, which establishes a thermo-current across the gap under closed-circuit condition. Even for devices constituted of homogeneous material, thermo-electric currents and voltages were systematically observed in similar device configurations [22], [23], [24, 36]. By taking these additional laser-induced thermal contributions into consideration, the photo-induced current *I*_phot_ may be written as a sum of several contributions depending on the laser intensity *P*_laser_ including one corresponding to the rectification of the optical field *I*_rect_, and others imparted by thermal effects such as electrode expansion, *I*_δd_, and thermo-current *I*_TE_. A variation of the optical potential *V*_opt_ and of the thermo-voltage *V*_TE_ will lead to a change of the total current dictated by the output characteristics *I*(*V*). The total current of an illuminated biased junction is thus

(9) $$\begin{array}{ll} I & =I_{\text{b}}+I_{\text{phot}}\\ & =I_{\text{b}}\left(V,d\right)+I_{\text{rect}}\left(V,V_{\text{opt}},d\right)\\ & \;+I_{\delta d}\left(P_{\text{laser}},d\right)+I_{\text{TE}}\left(V,\Delta T,d\right). \end{array}$$

For a given applied bias *V*, Eq. (9) clearly shows that a modification of the junction size *d* by the laser would affect all the contributing terms. This is therefore a sensitive contribution that needs to be taken into account.

## 3 Estimating the different contributions

In the following sections, we investigate the evolution of the different signals as a function of laser intensity and applied bias in order to identify the processes at play contributing to the measured currents.

### 3.1 Evolution of the tunnel current with laser intensity

**Figure 4:**
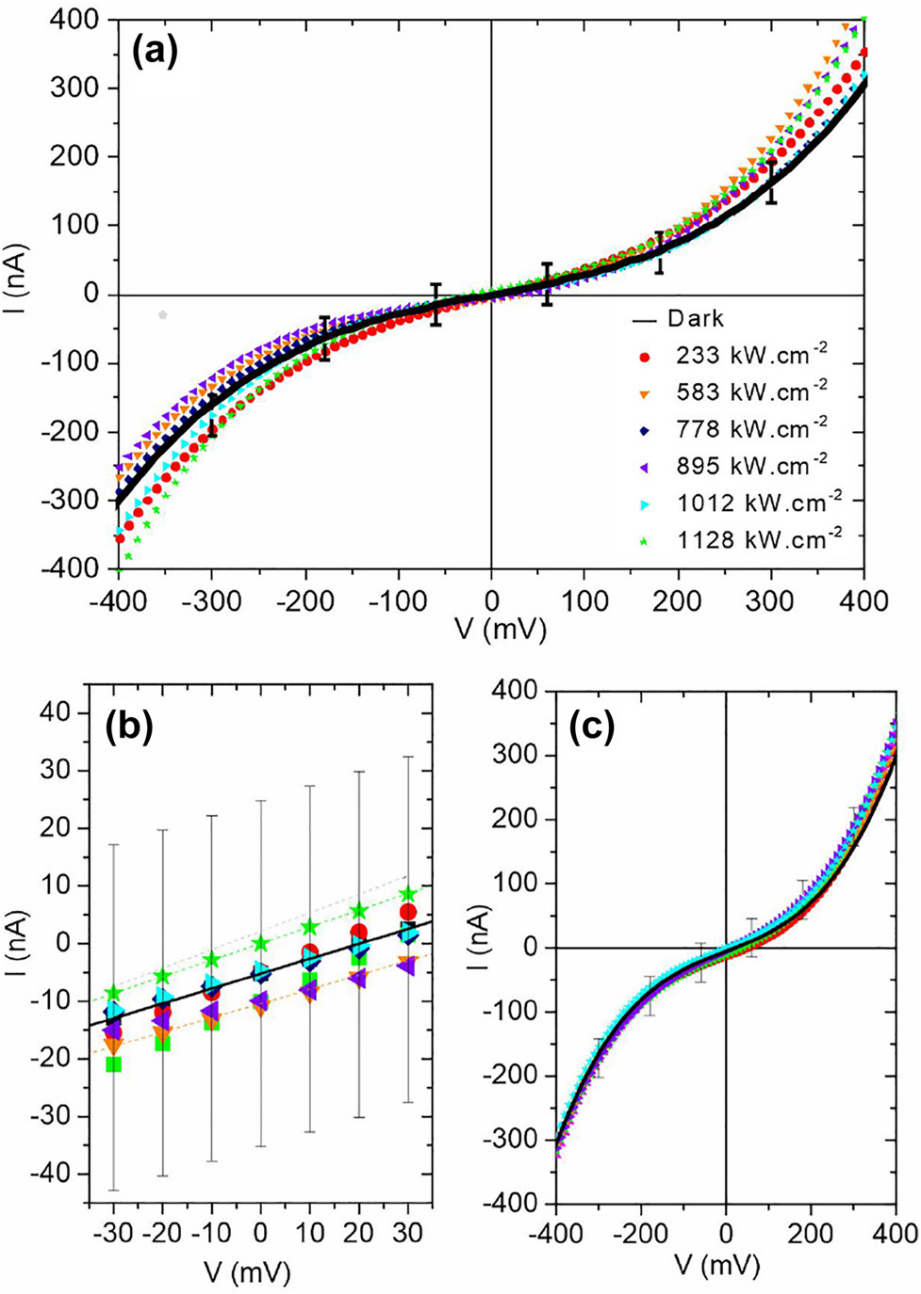
Electrical response of the rectenna upon irradiation. (a) *I*(*V*) characteristic of the junction under various laser intensity. (b) Zoom in of the curve around ±30 mV where the evolution of current evolves linearly with applied voltage. The dashed lines are linear fits to the data taken at different excitation intensities used to estimate the static conductance. (c) *I*(*V*) characteristics when the laser is positioned on one of the electrode, away from the junction.

In this section we interrogate how the total current contributing to Eq. (9) flowing through the junction is affected by the laser light. The output characteristic *I*(*V*) of the electromigrated junction measured under laser illumination is shown in Figure 4(a). The black solid line shows the output characteristic of the rectenna without any optical stimulus (dark response). The curves are running averages over the raw data. The level of noise on the signal can be appreciated by the error bars taken under dark condition. When illuminated, the deviation of the *I*(*V*) curve becomes increasingly pronounced with laser intensity but a clear evolution is hidden within the fluctuations. Nonetheless, this deviation with applied voltage can be placed in regard of the schematic depicted in Figure 3(b) where the magnitude of the rectified current increases with the nonlinearity of the *I*(*V*) curve. In the linear portion of the curve taken around the zero-bias condition (Figure 4(b)), the level of the noise also prevents us to establish a consistent trend with laser intensity in these measurements. A finer picture of the laser-induced signal will be presented in Section 3.2.

The evolution of the conductance and the nonlinearity of the output characteristic is a marker to assess the stability of the tunnel junction during the measurement. Increasing *V* or *P*_laser_ above the damage threshold will inevitably lead to a decrease of the conductance by a widening of the gap distance *d* and thereby changing the nonlinearity of the device. A first confirmation of the stability of the junction is demonstrated in Figure 4(b). The linear fits (dashed curves) to the data indicates that the static conductance stays constant with laser intensity at a mean value of 0.25 μS ± 5.3 ⋅ 10^−8^ S. A second demonstration is provided by the *I*(*V*) curves displayed in Figure 4(c) for various laser power. Here, the laser is placed on the upper electrode (Figure 2). There is no noticeable variation of the junction’s electrical characteristics upon the different voltage sweeps and laser powers sampled suggesting that the laser, and the voltage, do not affect the geometry of the device. This is further confirmed by the following measurements.

According to Simmon’s model of the transport (Eq. (3)), and in the limit of small voltage considered here, the exponential dependence of the tunneling current *I* does not depends on applied voltage *V* but is only driven by the size of the barrier *d*, and the effective work function Φ. This exponential dependence is conserved in the first derivative and subsequent derivatives of *I*(*V*). For the first and second derivative, the expressions read:

(10) $$\begin{array}{cc} \frac{dI}{dV} & =A\left(\frac{3.1\cdot 10^{-4}\sqrt{\Phi }}{d}+\frac{5.4\cdot 10^{-5}\Delta \Phi }{\Phi }V\right.\\ & \;\left.+\frac{1.65\cdot 10^{-4}d\sqrt{\Phi }}{\Phi }V^{2}\right)e^{-1.025d\sqrt{\Phi }}\\ \frac{d^{2}I}{dV^{2}} & =A\left(-\frac{5.4\cdot 10^{-5}\Delta \Phi }{\Phi }+\frac{3.3\cdot 10^{-4}d\sqrt{\Phi }}{\Phi }V\right)\\ & e^{-1.025d\sqrt{\Phi }} \end{array}$$

Experimentally, we measure the first and second derivative of the *I*(*V*) by a lock-in detection of the total current at the *n*th harmonic of the sync frequency of a 14 mV sinusoidal voltage *V*_AC_ applied to the rectenna [12, 14]. Demodulating a current at a frequency *n* ⋅ *f* is formally equivalent to a measure of the *n*th-derivative [37]. Here, *f* = 12.6 kHz. If *n* = 1, 2, the lock-in outputs *S* ^nf^ provide a magnitude of the junction’s dynamic conductance and nonlinearity and write:

(11) $$\begin{array}{cc} & S^{1\text{f}}=V_{\text{AC}}\frac{dI}{dV}\\ & S^{2\text{f}}=\frac{1}{4}V_{\text{AC}}^{2}\frac{d^{2}I}{dV^{2}} \end{array}$$

**Figure 5:**
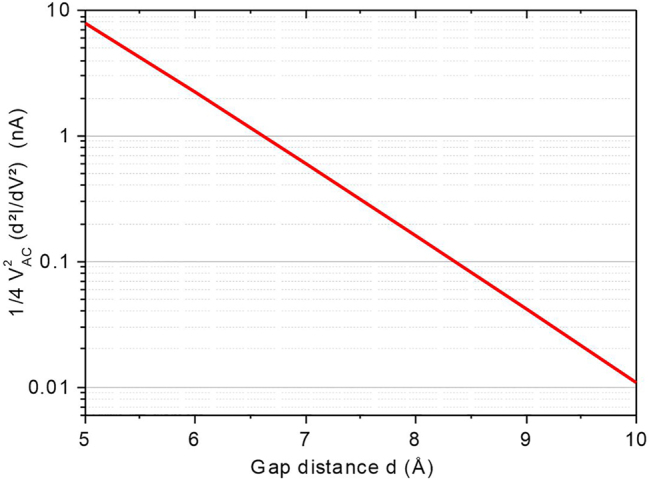
Semi logarithmic plot of the simulated evolution of the rectenna’s nonlinearity probed by the output of a lock-in detection with varied gap size *d*.

To appreciate how the nonlinearity of the *I*(*V*) curve depends on small variations of the gap size *d*, we plot in Figure 5 the simulated lock-in output *S* ^2f^ as a function of *d* for the junction parameters *A*, Φ, and ΔΦ deduced from the analysis of Figure 1(c) and using Eq. (10) and (11). We only consider a theoretical trend because we do not have the possibility to precisely control the gap size experimentally. We arbitrarily fix the applied voltage at *V* = 0.4 V. Clearly, a small change of the gap size resulting from a thermal expansion or an atomic rearrangement due to the laser excitation or the operating voltage would introduce a large modification of the current’ second derivative.

**Figure 6:**
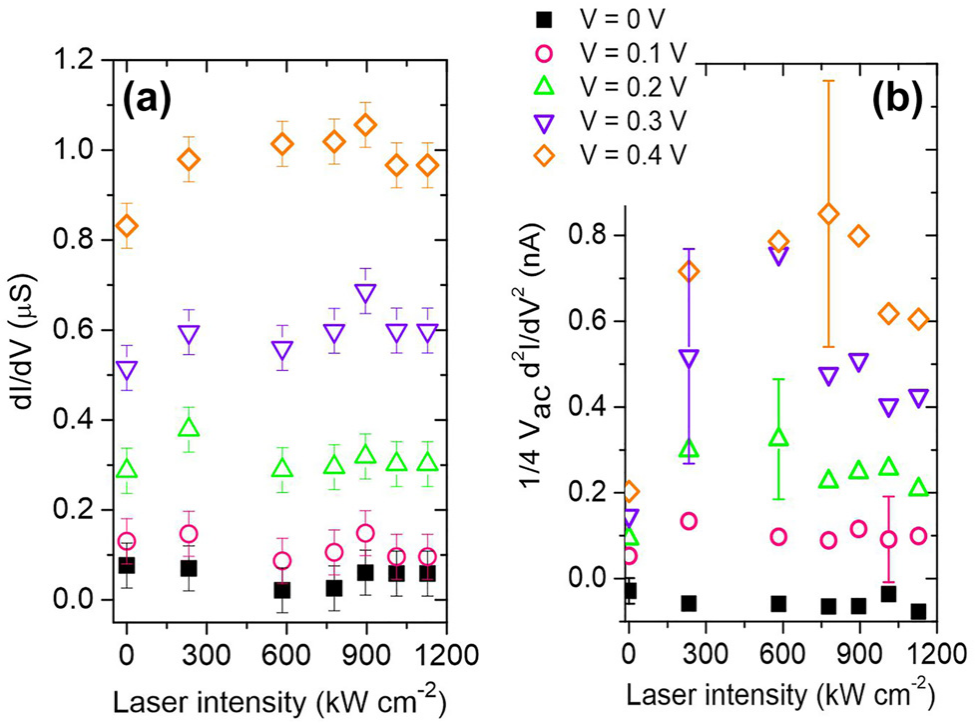
Evolution of the first (a) and the second derivatives (b) of the tunnel current measured by the lock-in with laser intensities for five bias set points *V*. (a) Measures the dynamic conductance of the junction while (b) provides its nonlinearity. The error bars represent the level of noise for a given dataset.

Figure 6(a) shows the dynamic conductance *G* of the junction experimentally determined by normalizing the lock-in output *S* ^1f^ with *V*_AC_ for different laser intensities and operating voltages *V*. Note that there is the slight discrepancy between the static conductance estimated from the fits of Figure 4(b) and the dynamic values because of the frequency-dependent impedance of the transimpedance amplifier. For the range of intensity interrogated, the dynamic conductance at null bias remains stable and confirms the static measurement performed by estimating the slopes of the *I*(*V*) in Figure 4(b). When increasing the applied bias, the dynamic conductance increases because the device enters its nonlinear regime. Here too, the dynamic conductance does not show sensible variations with laser intensity indicating that the junction’s intrinsic geometry is not altered by the illumination.

We confirm the stability against closing of the gap size by measuring the second derivative of the output characteristics with laser intensity shown in Figure 6(b). For *V* = 0 V, the junction’s response is linear and the non-linearity is approximately null. When operating the device with voltage set-points greater than 100 mV, the characteristic cannot be considered linear and the different terms in *V* ^2^ and *V* ^3^ in Eq. (3) are becoming predominant. Concomitantly, the lock-in output *S* ^2f^ registers a larger signal indicative of an increased nonlinearity. The point is that this quantity is fairly steady within the range of laser intensity sampled. The error bars are quantifying the level of fluctuations on the signal. It increases with bias but does not significantly changes with laser intensity. For the sake of clarity of the figure, only one error bar per voltage value is indicated. Compared to the trend expected if the laser would impart a linear expansion of the electrodes and a subsequent closing of the gap (Figure 5), we conclude that the dependence of the tunnel current with laser intensity observed in Figure 4 or the confocal response of Figure 2(b) cannot be accounted for a modified gap size *d* during illumination and voltage activation. This is probably because on the one hand the electrodes are firmly attached mechanically to the substrate’s surface with the help of the Cr adhesion layer, and on the other hand, the SiO_2_ acts as a thermal sink by dissipating the heat locally produced by the laser absorption [38].

The conservation of the intrinsic electrical properties indicates that thermal expansion of the electrodes is likely to be negligible in our measurement and thus *I*_*δ*d_ ≈ 0 in Eq. (9). The evolution of the total current with laser observed in the explored voltage range (Figure 4(a)) is then directly related to a current *I*_phot_ flowing through the rectenna and added to the tunnel current *I*_b_ imposed by the external potential.

### 3.2 Evolution of the photo-current *I*_phot_ with laser intensity

**Figure 7:**
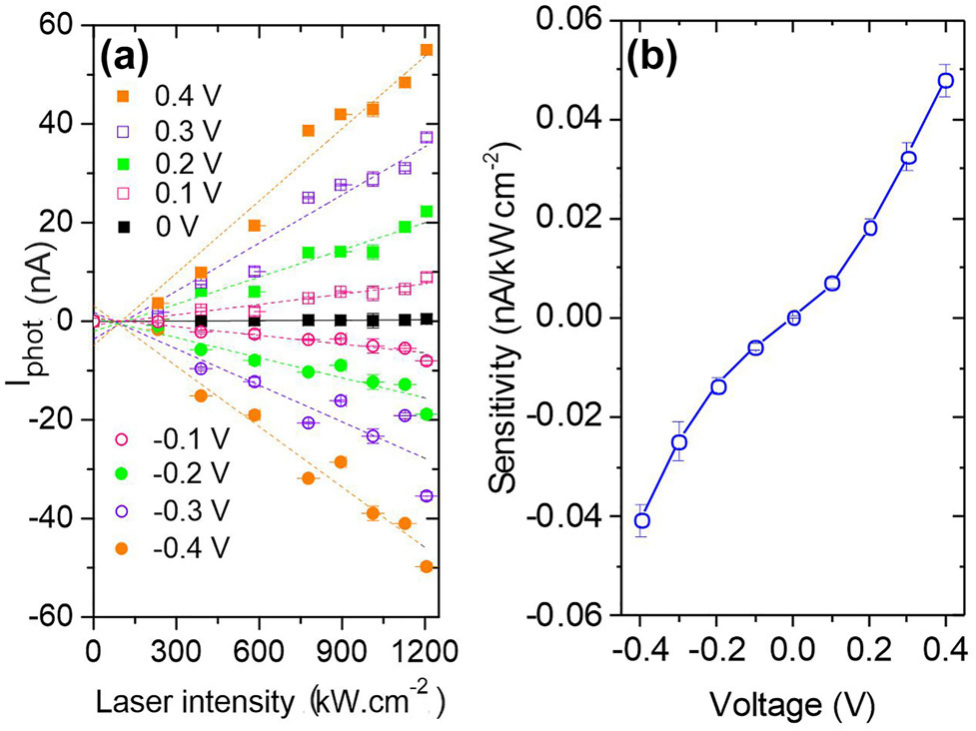
Photocurrent and sensitivity curves. (a) Laser-induced current contribution *I*_phot_ delivered by the rectenna as a function of laser intensity for various symmetric voltage set-points. The dotted lines are linear fits to the data. The slopes of the fits are reported in (b) for the corresponding voltages. The slope is a measure of the sensitivity of the device at a given bias set-point.

In the following paragraph we specifically study the sensitivity of the laser-induced contribution *I*_phot_ of the total current to the laser intensity. For this purpose, we interrogate the lock-in output synchronized at the chopper frequency (see Section 1.3) by changing the laser intensity and voltage set-points of the device. This measurement helps us to retrieve the trends hidden in the noise of the total current displayed in Figure 4. The results are displayed in Figure 7(a) and are represented by the data points. According to Eq. (9), *I*_phot_ is constituted of three different terms; one provided by the rectification of the optical field and the two others stemming from thermal effects. We concluded the previous section by a negligible contribution *I*_δd_. The remaining predominant terms in Eq. (9) are thus *I*_rect_ and *I*_TE_.

*I*_rect_ depends on the nonlinearity of the device and the square of the optical potential dropped across the gap $V_{\text{opt}}^{2}$ (Eq. (8)). In a first approximation *V*_opt_ is linked to the amplitude of the optical field via the relation *V*_opt_ = *E*_opt_ × *d*. This simple relation may be weighted by enhancement effect provided by the excitation of local surface plasmon resonances. In our experiments, we did not probe for such resonances as both polarization and laser wavelength are kept fixed. Because the measurements are averaged in space and time by the resolution of the microscope and the lock-in detection, any fluctuations due to the dynamic restructuring of the gap (atomic diffusion, cluster formation, …) are not detected. Nonetheless, the role of plasmonic enhancement may contribute to the device-to-device variability of the response. The laser intensity *P*_laser_ generates the optical field following the relation *P*_laser_ = *B*|*E*_opt_|^2^, where *B* is a proportionality factor. $V_{\text{opt}}^{2}$ is thus a linear function of the laser intensity with $V_{\text{opt}}^{2}\propto P_{\text{laser}}d^{2}$ . We can now understand the trend of *I*_phot_ featuring a linear dependency with *P*_laser_ in Figure 7(a) for all the operating voltages. The dotted lines are linear fits to the data. The fits are not constrained by a fixed intercept at *I*_phot_ = 0 because the data includes a dark noise contribution. The slope of the fits, which represents the sensitivity of the device, is reported in Figure 7(b) as a function of voltage *V*. As expected from Eq. (8), moving up the bias set-point increases the nonlinearity of the device and thus the term *d* ^2^ *I*/*dV* ^2^ (Figure 6(b)) and by consequence the rectified signal *I*_rect_.

**Figure 8:**
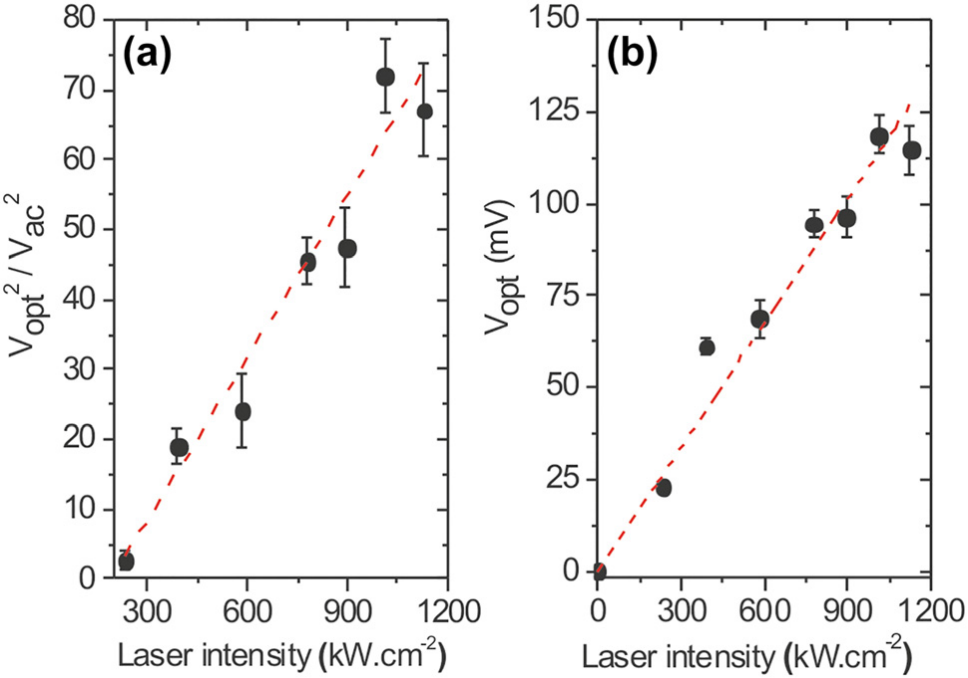
Optical potential produced by the rectenna. (a) Ratio $V_{\text{opt}}^{2}/V_{\text{AC}}^{2}$ with laser intensity. (b) Deduced values of *V*_opt_ with *V*_AC_ = 14 mV. The dashed lines in both graphs are linear fits. The linear trends are consistent with the rectification picture.

Comparing the magnitude of the signals *I*_phot_ = *I*_rect_ and *S* ^2f^ (Eq. (11)) enables to estimate the amplitude of *V*_opt_ generated by illuminating the gap with the laser. In Figure 8(a), we make the ratio between $I_{\text{phot}}/S^{2f}=V_{\text{opt}}^{2}/V_{\text{AC}}^{2}$ . *V*_AC_ is fixed experimentally at 14 mV. Here too, the ratio has a clear linear relationship with the laser intensity. Figure 8(b) shows the resulting optical potential *V*_opt_ as a function of the laser intensity. The inferred values are consistent with earlier a report of Ward et al. [12] and are within the validity of the semi-classical description of the rectification process since *eV*_opt_ ≪ *ℏω*. In this study, the step-like evolution of the *I*(*V*) upon the rectification process is not observed since the nonlinearity of the junction in the scale of *V*_opt_ is not sufficient to identify a quantized rectified current. The photo-assisted tunneling current can thus be described from a classical formalism. Clearly, the trends observed in Figure 7 and in Figure 8 are fully in line with rectification picture, and the thermo-current contribution *I*_TE_ seems to be negligible.

Any significant thermal component of the current would manifest itself by a deviation of the linear behavior registered in Figure 7(a). We stress that all the above measurements were acquired a fixed laser focused and centered on the junction. In this case, the temperature raise on both sides can be considered symmetric as the size of the diffraction-limited focal region (ca. 260 nm) mostly overlaps with the metal region forming the tapered electrodes. We conclude that in this particular device, local structural changes (e.g. atomic-size defects) of the gap (Figure 1(b)) are not contributing to unbalance temperature between the two sides of the gap when the laser is centered. This is in line with the results of Ref. [20] for which a vanishing thermovoltage was observed even in the presence of electrochemical deposits. Of course, this is no longer true when the excitation does not evenly excite the junction. In Ref. [22], a clear spatial dependence of thermo-voltage amplitude and polarity were observed. We note that the confocal image in Figure 2(a) showing the back-scattered laser signal does not reveal any local resonant enhancing site in the gap that can be related to the specificity of the gap geometry.

### 3.3 Example of a thermal effect

**Figure 9:**
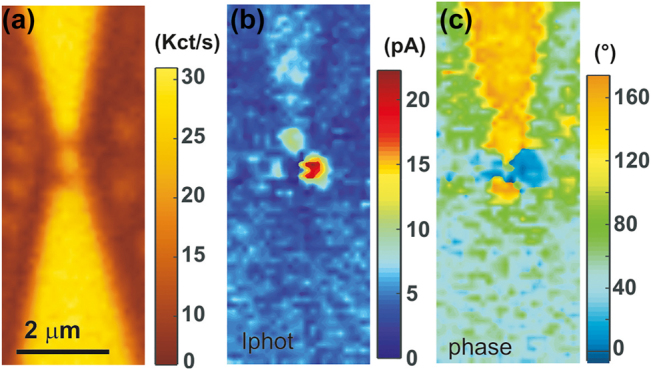
Evidence of thermal effects in spatially resolved confocal maps. (a) Map of the back-scattered laser intensity. The rounded features nearby the tapered sections are gold nanoparticles used in another study  (b) and (c) are maps showing the simultaneously measured modulus |*I*_phot_| and phase *ζ* of the laser-induced current. *V* = 0 V and *P*_laser_ = 1.09 MW cm^2^.

In the previous sections, we argued that the thermal imbalance between the two metal electrodes can be neglected on account of a symmetric excitation, *i.e*. a laser focused on the rectifying feed. While this was true with the junction discussed above (see for instance the confocal maps of Figure 2), the intricacy of the electromigration process and the resulting gap geometry sometimes lead to more complicated case with clear evidence of a current flow *I*_TE_ (Eq. (9)) generated by a thermal gradient. Such an example is illustrated in the confocal maps of Figure 9. The outline of the device is pictured in the back-scattered laser intensity map of Figure 9(a) taken at a reduced laser power. With the diffraction-limited resolution of the microscope, there is no significant difference between this device and the previous junction’s confocal response (Figure 2(a)). However, the feedgaps are likely to be significantly different because of the stochastic evolution of the electromigrated process (see for instance Figure 1(b)). The photo-current map of Figure 9(b) indeed features a drastically different behavior. The power of the laser is here higher than in Figure 2 with *P*_laser_ = 1089 kW cm^−2^. Two distinct spots, one intense and a second one less pronounced, are now dictating the spatial response of the device. A weak photo-response is also detected when the laser impinges on the upper electrode, but this response is not seen when the laser hits the lower electrode. Such signal from the access electrode was also not observed with the device logged in Figure 2. This photo-current generated when the laser shines on an electrode is a signature of a thermo-current flowing in the device [23] created by laser-induced temperature difference between the two leads. At that stage we do not have a reasonable explanation for the vanishing *I*_TE_ when the lower electrode is illuminated, but such asymmetric response was observed in biased constriction when heat flux opposes to the current [23]. The phase map of Figure 9(c) confirms the thermal nature of the photo-current. There is a net and abrupt ≈170° phase shift between the principal features in the *I*_phot_ map indicating a direction of the current which depends on the precise location of the laser with respect to the symmetry of the device. Thus, the rectifying response of two devices is seemingly indistinguishable from the diffraction-limited confocal maps behave differently upon laser excitation of their respective feed-gap. This is probably linked to the particularity of the electrodes’ geometry defining the electrical and optical feedgap of the rectenna.

## 4 Conclusions

To conclude, we reviewed and identified the different origins of the current photo-generated by electromigrated planar optical rectennas. Because this new family of devices consists of a tunneling junction formed between two metal electrodes, thermal effects inherent to light absorption must be considered. This is a key aspect to consider as the intrinsic dynamics behind optical rectification and thermal effects are differing by many orders of magnitude.

The differentiation of thermal processes from optical rectification phenomenon was carried out by electronic characterisation of the rectifying feed under direct laser illumination. Tracking of the junction’s dynamic conductance and the second derivative of its output characteristic enabled us to exclude any possibility of thermal expansion of the metal electrodes. The rectenna intrinsic electrical characteristics are thus maintained throughout the experiment thanks to an efficient mechanical attachment of the electrodes to the substrate, which also acts as a heat sink.

The extraction of photo-current *I*_phot_ from the total electrical current generated by the device under a symmetrical illumination was performed by lock-in detection. We have shown that a linear evolution of the photo-current with the laser intensity indicates that the measured photo-current is a rectified contribution produced by the device. The ratio of the photo-current signal and the electrical nonlinearity of the rectenna allowed us to infer the optical voltage *V*_opt_ dropped at the rectenna’s feed. We found values of a few tens of mV in agreement with earlier reports. Considering these low *V*_opt_, the rectified current can be accurately described by classical rectification formalism. The nonlinearity of the output characteristics at the scale of a few tens of millivolts is not sufficient to observe a quantization of the rectified current as expected by the semiclassical model usually used for high frequencies excitation.

We found that asymmetric illumination of the junction might cause the establishment of a thermal gradient. This affects the tunnel barrier electronic distribution due to a thermal imbalance between the two metal electrodes. An additional current is then generated and added on the rectified signal. The direction of this thermo-induced current flow can be deduced by extracting the phase of the measured photo-current. The current drifts according to the illuminated side of the junction. The fine details of the metal terminations are thus likely to determine the weight between the different physical mechanisms contributing to the laser-induced current. From the technological point of view, engineering the gap environment at this length scale remains a formidable challenge, but a prerequisite to the development of optical rectennas.

## References

[1] Miskovsky N. M., Cutler P. H., Mayer A. (2012). Nanoscale devices for rectification of high frequency radiation from the infrared through the visible: a new approach. J. Nanotechnol..

[2] Moddel G., Grover S. (2013). Rectenna Solar Cells.

[3] Rechberger W., Hohenau A., Leitner A., Krenn J. R., Lamprecht B., Aussenegg F. R. (2003). Optical properties of two interacting gold nanoparticles. Opt. Commun..

[4] García-Martín A., Ward D. R., Natelson D., Cuevas J. C. (2011). Field enhancement in subnanometer metallic gaps. Phys. Rev. B.

[5] Viljas J. K., Cuevas J. C. (2007). Role of electronic structure in photoassisted transport through atomic-sized contacts. Phys. Rev. B.

[6] Mayer A., Cutler P. H. (2009). Rectification properties of geometrically asymmetric metal-vacuum-metal junctions: a comparison of tungsten and silver tips to determine the effects of polarization resonances. J. Phys. Condens. Matter.

[7] Février P., Gabelli J. (2018). Tunneling time probed by quantum shot noise. Nat. Commun..

[8] Faris S. M., Fan B., Gustafson T. K. (1975). Electronic tunneling currents at optical frequencies. Appl. Phys. Lett..

[9] Tucker J. (1979). Quantum limited detection in tunnel junction mixers. IEEE J. Quantum Electron..

[10] Reynaud C. A., Duché D., Simon J. J. (2020). Rectifying antennas for energy harvesting from the microwaves to visible light: a review. Prog. Quantum Electron..

[11] Ittah N., Noy G., Yutsis I., Selzer Y. (2009). Measurement of electronic transport through 1g0 gold contacts under laser irradiation. Nano Lett..

[12] Ward D. R., Hüser F., Pauly F., Cuevas J. C., Natelson D. (2010). Optical rectification and field enhancement in a plasmonic nanogap. Nat. Nanotechnol..

[13] Arielly R., Ofarim A., Noy G., Selzer Y. (2011). Accurate determination of plasmonic fields in molecular junctions by current rectification at optical frequencies. Nano Lett..

[14] Stolz A., Berthelot J., Mennemanteuil M. M. (2014). Nonlinear photon-assisted tunneling transport in optical gap antennas. Nano Lett..

[15] Hobbs P. C. D., Laibowitz R. B., Libsch F. R., LaBianca N. C., Chiniwalla P. P. (2007). Efficient waveguide-integrated tunnel junction detectors at 1.6 μm. Opt. Express.

[16] Du W., Wang T., Chu H. S., Nijhuis C. A. (2017). Highly efficient on-chip direct electronic–plasmonic transducers. Nat. Photonics.

[17] Dasgupta A., Mennemanteuil M. M., Buret M., Cazier N., Colas-des-Francs G., Bouhelier A. (2018). Optical wireless link between a nanoscale antenna and a transducing rectenna. Nat. Commun..

[18] Grafström S. (2002). Photoassisted scanning tunneling spectroscopy. J. Appl. Phys..

[19] Shi S. F., Xu X., Ralph D. C., McEuen P. L. (2011). Plasmon resonance in individual nanogap electrodes studied using graphene nanoconstrictions as photodetectors. Nano Lett..

[20] Kopp B., Yi Z., Benner D. (2012). Revealing thermal effects in the electronic transport through irradiated atomic metal point contacts. Beilstein J. Nanotechnol..

[21] Ofarim A., Kopp B., Möller T. (2016). Thermo-voltage measurements of atomic contacts at low temperature. Beilstein J. Nanotechnol..

[22] Zolotavin P., Evans C., Natelson D. (2017). Photothermoelectric effects and large photovoltages in plasmonic au nanowires with nanogaps. J. Phys. Chem. Lett..

[23] Mennemanteuil M. M., Colas-des-Francs G., Buret M. (2018). Laser-induced thermoelectric effects in electrically biased nanoscale constrictions. Nanophotonics.

[24] Tsutsui M., Morikawa T., Arima A., Taniguchi M. (2013). Thermoelectricity in atom-sized junctions at room temperatures. Sci. Rep..

[25] Lee W., Kim K., Jeong W. (2013). Heat dissipation in atomic-scale junctions. Nature.

[26] Kim Y., Ang C. H., Ang K., Chang S. W. (2021). Electromigrated nanogaps: a review on the fabrications and applications. J. Vac. Sci. Technol. B.

[27] Dasgupta A., Buret M., Cazier N. (2018). Electromigrated electrical optical antennas for transducing electrons and photons at the nanoscale. Beilstein J. Nanotechnol..

[28] Simmons J. G. (1963). Generalized formula for the electric tunnel effect between similar electrodes separated by a thin insulating film. J. Appl. Phys..

[29] Brinkman W. F., Dynes R. C., Rowell J. M. (1970). Tunneling conductance of asymmetrical barriers. J. Appl. Phys..

[30] Mangin A., Anthore A., Della Rocca M. L., Boulat E., Lafarge P. (2009). Transport through metallic nanogaps in an in-plane three-terminal geometry. J. Appl. Phys..

[31] Kern J., Kullock R., Prangsma J. C., Emmerling M., Kamp M., Hecht B. (2015). Electrically-driven optical antennas. Nat. Photonics.

[32] Frimmer M., Puebla-Hellmann G., Wallraff A., Novotny L. (2014). The role of titanium in electromigrated tunnel junctions. Appl. Phys. Lett..

[33] Tu X. W., Lee J. H., Ho W. (2006). Atomic-scale rectification at microwave frequency. J. Chem. Phys..

[34] Bragas A. V., Landi S. M., Martínez O. E. (1998). Laser field enhancement at the scanning tunneling microscope junction measured by optical rectification. Appl. Phys. Lett..

[35] Tien P. K., Gordon J. P. (1963). Multiphoton process observed in the interaction of microwave fields with the tunneling between superconductor films. Phys. Rev..

[36] Abbasi M., Evans C. I., Chen L., Natelson D. (2020). Single metal photodetectors using plasmonically-active asymmetric gold nanostructures. ACS Nano.

[37] Adler J. G., Jackson J. E. (1966). System for observing small nonlinearities in tunnel junctions. Rev. Sci. Instrum..

[38] Mennemanteuil M. M., Buret M., Cazier N. (2016). Remote plasmon-induced heat transfer probed by the electronic transport of a gold nanowire. Phys. Rev. B.

